# Necrotizing fasciitis following laparoscopic total extra peritoneal repair of left inguinal hernia

**DOI:** 10.4103/0972-9941.30683

**Published:** 2007

**Authors:** Vishwanath Golash

**Affiliations:** Department of Surgery, Sultan Qaboos Hospital, Salalah, Sultanate of Oman

**Keywords:** Inguinal hernia, laparoscopy, necrotizing fasciitis

## Abstract

There are rare reports of necrotizing fasciitis (NF) following laparoscopic surgery. The clinical presentation of this condition may be delayed due to non-specific symptoms and sign. The diagnosis is essentially clinical and early recognition is crucial in the management. We present a case of NF of the lower abdominal wall extending to thigh, scrotum and perianal area following the laparoscopic extraperitoneal repair of left inguinal hernia managed with extensive debridment, removal of mesh, antibiotic, and skin grafting. He was seen 6 months after his surgeries and had no disability. The extensive search on Medline, Medscape, and Google engine revealed only one case report of NF following laparoscopic total extraperitoneal repair of inguinal hernia that died and this is the second case report and the only surviving one.

## INTRODUCTION

Necrotizing fasciitis (NF) is a life threatening rapidly spreading soft tissue bacterial infection, progressing to extensive necrosis and gangrene of subcutaneous tissue and fascia. It is a deep-seated infection often mistaken as simple cellulitis around the wounds but the intensity of pain and toxicity is out of proportion to the local findings. Quite often the diagnosis is made in the presence of obvious signs. The lower abdominal wall and the perineum are by far the most affected parts of the body. The etiology of NF after surgery is polymicrobial in the majority of the patient and its progression is slower than that seen in monomicrobial infections. Gas in subcutaneous tissue is usually not a feature of polymicrobial NF after the surgery. There are several comorbid conditions, which can make the patients more susceptible due to lowered immunity such as contaminated operation, trauma, diabetes, and immunosuppression. The mortality is high when diagnosis and debridment is delayed.

## CASE REPORT

A 41 years old male patient previously healthy, presented with the history of laparoscopic repair of left inguinal hernia in a neighboring country 12 days ago. The patient noticed pain and redness around the port sites five days after the operation and reported to his doctor. He was given a course of antibiotics for one week but his redness, pain and swelling spread further on abdomen wall, thighs and scrotum. Two days prior to admission he was in severe pain, unable to move and had chills with rigor. He had nausea and loss of appetite. There was no known history suggestive of any pre-disposing- associated comorbid conditions.

### On examination:

**Figure 1:**
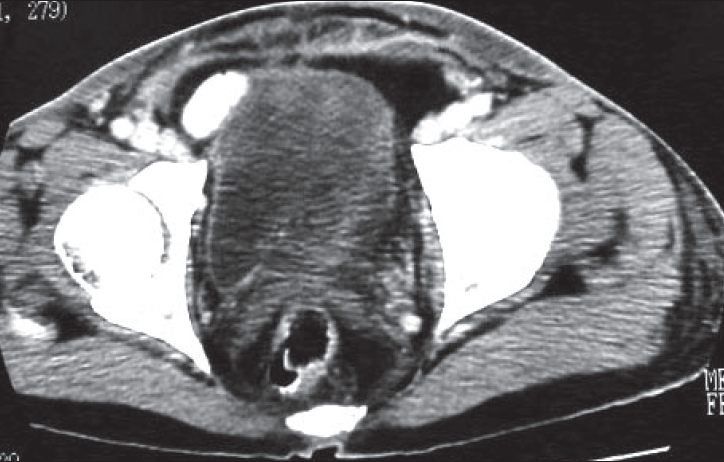
CT scan of the lower abdomen

**Figure 2:**
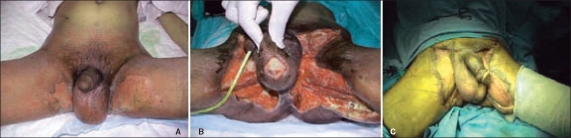
A. Extensive celluliitis, B. Debridement, C. Skin grafting

He was toxic, tachycardic, emaciated, and febrile. The cellulitis and tense edema with tenderness was noticed involving the whole of the lower abdomen, scrotum, perineum, and the upper medial aspects of thighs. Skin blistering and ruptured bullae with foul smelling serous discharge were seen on the inner thighs [Figure 2A]. No crepitus was felt in the affected area. His CT scan showed extensive inflammation of the subcutaneous tissues of anterior abdominal wall and thighs, fluid around the spermatic cords, collection medial to left obturator muscle, free gas in the subcutaneous planes. No extension of inflammation and fluid collection was seen in the retroperitoneum [Figure 1]. His laboratory parameters were as follow: White cell count: 19.7; Hemoglobin, 11.1 gm; Random blood sugar, 5.37 mmols; Urea, 7.19; Creatinine, 53.8; Na, 130. His liver and kidney functions were normal and was HIV negative. Blood cultures were taken. The clinical and radiological findings were diagnostic of NF.

He was resuscitated with IV fluids, oxygen, and parental broad-spectrum antibiotics, and was taken up for urgent surgical exploration. The diagnosis was established intra-operatively. Through a long left transverse inguinal incision the tissue planes were opened. The mesh was lying loose and infected and was removed. There was offensive smelling pus in all tissue planes sparing muscles. The incisions were extended further and all dead and necrotic tissues were excised [Figure 2B]. He had several excisions of necrosed tissues over the course of one month. The blood and wound cultures grew *Escherichia Coli*, Coliform, Klebsiella, *Proteus Mirabilis*, and *Staphylococcus Aureus*. A split skin grafting was done to cover the exposed area and muscles [Figure 2C]. No disability and recurrence of hernia was seen 6 months after the surgeries.

## DISCUSSION

With the development of laparoscopic approached the NF is considered a rare complication. There are only few case reports in the literature of NF after laparoscopic surgery. Although rare, the mortality is very high and death secondary to NF in laparoscopic surgery has been reported in unrecognized strangulated lateral trocar hernia, peritoneal contamination with intestinal contents and following total extraperitoneal repair of inguinal hernia.[1–3] Etiology is unknown but possible causes are contamination of instruments and abdominal wall at trocar sites with pathological organisms and injury to bowel.[4] Primary infection of mesh leading to NF was another possibility in this patient. The gas in tissue planes or discoloration of skin is not a universal finding and this misconception can delay the differential diagnosis from cellulitis.[5] This patient was taking antibiotics for last one week prior to his admission and this may have delayed his presentation. The fine needle aspiration for culture, tissue biopsy, CT, and MRI may be useful in diagnosis. In this patient CT was useful in excluding the extension of NF in retroperitoneum. All the signs may not be there and in majority the diagnosis is intraoperative. Early diagnosis, radical excisions of necrosed tissue and antibiotics are the standard treatment. Secondary surgical procedure like diversion colostomy, amputations, skin grafting, and flap may be needed.
